# Effect of Convalescent Plasma in Critically Ill Patients With COVID-19: An Observational Study

**DOI:** 10.3389/fmed.2021.630982

**Published:** 2021-01-28

**Authors:** Pedro Kurtz, Cassia Righy, Monica Gadelha, Fernando A. Bozza, Patricia T. Bozza, Bruno Gonçalves, Leonardo S. L. Bastos, Andre M. Vale, Luiza M. Higa, Leda Castilho, Fabio L. Monteiro, Nestor Charris, Fernanda Fialho, Ricardo Turon, Alexandro Guterres, Renan Lyra Miranda, Carlos Henrique de Azeredo Lima, Vanessa de Caro, Marco Aurelio Prazeres, Nina Ventura, Clara Gaspari, Fabio Miranda, Paulo Jose da Mata, Margarida Pêcego, Sheila Mateos, Maria Esther Lopes, Shirley Castilho, Álvaro Oliveira, Carla Boquimpani, Andréa Rabello, Josiane Lopes, Orlando Conceição Neto, Orlando da C. Ferreira, Amilcar Tanuri, Paulo Niemeyer Filho, Luiz Amorim

**Affiliations:** ^1^Instituto Estadual do Cérebro Paulo Niemeyer, Rio de Janeiro, Brazil; ^2^D'Or Institute for Research and Education, Rio de Janeiro, Brazil; ^3^National Institute of Infectious Disease Evandro Chagas, Oswaldo Cruz Foundation, Rio de Janeiro, Brazil; ^4^Laboratory of Immunopharmacology, Oswaldo Cruz Institute, Oswaldo Cruz Foundation, Rio de Janeiro, Brazil; ^5^Industrial Engineering Department, Pontifical Catholic University of Rio de Janeiro (PUC-Rio), Rio de Janeiro, Brazil; ^6^Laboratory of Lymphocyte Biology, Program in Immunobiology, Carlos Chagas Filho Institute of Biophysics, Federal University of Rio de Janeiro (UFRJ), Rio de Janeiro, Brazil; ^7^Laboratory of Molecular Virology, Department of Genetics, Institute of Biology, Federal University of Rio de Janeiro, Rio de Janeiro, Brazil; ^8^Laboratory of Cell Culture Engineering, COPPE, Chemical Engineering Program, Federal University of Rio de Janeiro (UFRJ), Rio de Janeiro, Brazil; ^9^Instituto Estadual de Hematologia Arthur de Siqueira Cavalcanti (HEMORIO), Rio de Janeiro, Brazil

**Keywords:** convalescent plasma, coronavirus, acute respiratory distress syndrome, COVID-19, survival

## Abstract

**Background:** Convalescent plasma is a potential therapeutic option for critically ill patients with coronavirus disease 19 (COVID-19), yet its efficacy remains to be determined. The aim was to investigate the effects of convalescent plasma (CP) in critically ill patients with COVID-19.

**Methods:** This was a single-center prospective observational study conducted in Rio de Janeiro, Brazil, from March 17th to May 30th, with final follow-up on June 30th. We included 113 laboratory-confirmed COVID-19 patients with respiratory failure. Primary outcomes were time to clinical improvement and survival within 28 days. Secondary outcomes included behavior of biomarkers and viral loads. Kaplan–Meier analyses and Cox proportional-hazards regression using propensity score with inverse-probability weighing were performed.

**Results:** 41 patients received CP and 72 received standard of care (SOC). Median age was 61 years (IQR 48–68), disease duration was 10 days (IQR 6–13), and 86% were mechanically ventilated. At least 29 out of 41CP-recipients had baseline IgG titers ≥ 1:1,080. Clinical improvement within 28 days occurred in 19 (46%) CP-treated patients, as compared to 23 (32%) in the SOC group [adjusted hazard ratio (aHR) 0.91 (0.49–1.69)]. There was no significant change in 28-day mortality (CP 49% vs. SOC 56%; aHR 0.90 [0.52–1.57]). Biomarker assessment revealed reduced inflammatory activity and increased lymphocyte count after CP.

**Conclusions:** In this study, CP was not associated with clinical improvement or increase in 28-day survival. However, our study may have been underpowered and included patients with high IgG titers and life-threatening disease.

**Clinical Trial Registration:** The study protocol was retrospectively registered at the Brazilian Registry of Clinical Trials (ReBEC) with the identification RBR-4vm3yy (http://www.ensaiosclinicos.gov.br).

## Background

As of October 12th, 2020, the coronavirus disease 2019 (COVID-19) pandemic has affected more than 37 million people worldwide. Brazil is amongst the hardest hit countries, with more than 5 million confirmed cases and over 150,000 deaths (1). Published studies of large cohorts show that, in critically ill patients, mortality has ranged from 39.5 to 54% (2–4). Although evidence-based management of patients with severe COVID-19 in the intensive care unit is evolving rapidly (5, 6), mortality remains high. Convalescent plasma (CP) is a potential therapeutic option for COVD-19. Preliminary reports of severe patients with suspected or confirmed severe acute respiratory syndrome coronavirus 2 (SARS-CoV-2) suggested improvement in their viral load, laboratory markers of inflammation and organ dysfunction (7–13). A randomized trial of CP that enrolled patients with severe and life-threatening COVID-19 was conducted in Wuhan, China, but was stopped early due to slow enrollment after the containment of the epidemic (14). Albeit the primary outcome of time to clinical improvement was not different between groups, the subgroup of patients requiring oxygen support without mechanical ventilation benefited from CP therapy. A subsequent randomized trial from India showed no benefit of CP in the combined outcomes of disease progression or all-cause mortality at 28 days in a cohort where the majority of patients had neutralizing antibodies prior to the treatment (15). Thus, the data available do not support the use of CP for moderate or severe SARS-CoV-2 infection (16). However, due to positive results in subgroups and limitations in published studies, whether CP is beneficial in specific populations of COVID-19 patients remains uncertain.

The aim of the present observational study was to investigate the effect of convalescent plasma therapy in the clinical improvement and 28-day mortality of critically ill patients with COVID-19.

## Methods

### Study Design and Patients

This was a prospective observational study conducted at the Instituto Estadual do Cérebro Paulo Niemeyer (IECPN). Starting on March 17th, 2020, all patients admitted to the ICU with suspected COVID-19 underwent diagnostic testing for SARS-CoV-2 through either nasopharyngeal swabs or tracheal aspirates, when intubated. A reverse transcriptase-polymerase chain reaction (RT-PCR) assay was performed with a turnaround time of <12 h. All patients admitted to our ICU received usual standard of care (SOC) for severe COVID-19 (confirmed disease and need of oxygen support) and were included in a cohort study that recorded daily clinical and laboratory data in a dedicated case report form^1^. The Convalescent Plasma (CP) observational study protocol was approved on April 17th. For the purposes of the comparisons presented in this manuscript, patients in the SOC group were those admitted to our ICU between March 17th (first case admitted) and April 17th. Between April 18th and May 30th, all consecutive adult patients (age > 18 years) with suspected or confirmed SARS-CoV-2 infection admitted to the ICU or intubated for COVID-19-related respiratory failure for up to 3 days were considered eligible to receive CP therapy. Patients with negative RT-PCR for SARS-CoV-2 and/or life expectancy <24 h, both in the SOC and CP groups, were excluded from the analysis (Supplementary eFigure 1). Eligible patients with confirmed COVID-19 and treated with CP between April 18th and May 30th were compared to patients that received only SOC treatment. Follow-up continued through June 30th, or a minimum of 28 days for all patients.

The study protocol was approved by the National Ethics Committee and the Institutional Review Boards (IRB) of the Instituto Estadual de Hematologia Arthur de Siqueira Cavalcanti (HEMORIO) and of the IECPN (3.976.630). The study protocol was registered at the Brazilian Registry of Clinical Trials (ReBEC) with the identification RBR-4vm3yy. Informed consent was obtained from all patients' representatives.

### Procedures and Data Collection

All patients admitted to the ICU received SOC management, which consisted of respiratory supportive therapy with either oxygen through a non-rebreather mask or mechanical ventilation (MV). At the time of our study there were no published randomized trials supporting the use of steroids for patients with viral pneumonia and/or acute respiratory distress syndrome (ARDS) related to COVID-19. Hydrocortisone for refractory septic shock and methylprednisolone or dexamethasone for ARDS were used according to the decision of the attending physicians. Moreover, all patients received prophylactic anticoagulation with enoxaparin (40 to 60 mg daily), unless contraindicated. Full therapeutic anticoagulation was reserved for patients with clinical evidence of thromboembolism. Patients under invasive ventilation, with and without ARDS, were managed with a protective ventilation strategy that included low tidal volume (6 mL/kg of predicted body weight) and driving pressure (<16 cmH2O) as well as optimal PEEP. Best PEEP was calculated based on the best lung compliance and PaO2/FiO2 ratio. In those with moderate to severe ARDS and PaO2/FiO2 ratio below 150, despite neuromuscular blockade and optimal ventilatory settings, prone position was initiated.

The first infusion of 200 to 250 mL of ABO-compatible previously frozen CP was administered up to 3 days after ICU admission or respiratory failure and endotracheal intubation. After preliminary analyses of the first 10 treated patients, authors observed a secondary rise in C reactive protein levels and persistent viral loads, which lead to a modification of the clinical protocol, approved by the IRB, including a second infusion within the first week in subsequent patients.

Clinical and laboratory data were recorded at admission and sequentially for all patients included in the study for up to 14 days. In patients treated with CP, blood and respiratory secretion samples were obtained on baseline and after 1, 3, and 7 days for assessment of viral load and concentrations of inflammatory cytokines.

### Convalescent Plasma Safety

Intervention safety was monitored through careful patient surveillance, focused on CP adverse effects, at the beginning and at the completion and 24 h after CP transfusion. The transfusion medicine team checked for allergic reactions, Transfusion-Associated Circulatory Overload (TACO), Transfusion-Related Acute Lung Injury (TRALI), Pro-Thrombotic effects and acute hemolysis.

### Molecular, Immunological and Cytokine Assessments

Before and after CP therapy, respiratory tract and blood samples were collected, and SARS-CoV-2 RNA was evaluated through quantitative RT-PCR (RT-qPCR). All serial samples were stored at −80°C and then processed at a single time. Cycle Threshold (Ct) values were obtained on baseline, days 1, 3, and 7 after CP therapy. Endpoint titers of IgG anti-SARS-CV-2 S protein were measured in all donors and CP recipients using a homebrew ELISA test (for details see Supplementary Material). In order to examine inflammation and immune activation, we performed a multiplex immunoassay containing fluorescent dyed microbeads for plasma cytokine measurements of interleukin (IL)-6 and IFN-γ-induced protein 10 (IP-10).

### Outcomes

The primary outcomes of our study were the time to clinical improvement and survival within 28 days. The former was defined by a 2-point reduction from patients' admission status on a 10-point ordinal scale that was evaluated daily (17) or discharge from the hospital, whichever came first. The clinical scale applied ranges from 1 to 10, where values below 4 mean that the patient is ambulatory (i.e., discharged from the hospital) and 10 that the patient died. Values from 4 to 9 represent an increasing need of oxygen and respiratory support, as follows: (4) hospitalized, no oxygen support; (5) hospitalized, oxygen by mask or nasal prongs; (6) hospitalized, oxygen by non-ivasive ventilation or high flow nasal cannula; (7) intubated and mechanically ventilated, PO2/FiO2≥150; (8) mechanical ventilation and PO2/FIO2 <150 or vasopressors; (9) mechanical ventilation with PO2/FiO2 <150 and vasopressors, dialysis, or extracorporeal membrane oxygenation (ECMO). Patients that died within 28 days were considered as having no clinical improvement. Secondary outcomes analyzed included the behavior of biomarkers in treated and untreated patients.

## Statistical Analysis

We reported categorical variables as number (%) and continuous variables as median and interquartile range (IQR). Comparisons between groups were performed using the chi-square test and the Mann–Whitney *U*-test for categorical and continuous variables, respectively.

We compared patients treated with CP with patients treated with SOC. To help account for the non-randomized administration of CP, we used propensity-score weighting methods to reduce the effects of confounding. The individual propensities for receipt of CP were estimated in all patients with the use of a multivariable logistic-regression model that included the following clinical covariates, based on clinical grounds: Age, Sex, Simplified Acute Physiology Score (SAPS 3) (18), Sequential Organ Failure Assessment (SOFA) score (19) score, mechanical ventilation on admission and days from symptom onset to ICU admission. Then, univariate Kaplan–Meier curves and Cox proportional-hazards regression models were used to estimate the association between CP therapy and two separate outcomes: (1) death at 28 days; (2) clinical improvement within 28 days, as defined by a 2-point reduction from patients' admission status on a 10-point ordinal scale (17), or discharge from the hospital, whichever came first. Associations between CP use and the outcomes described above were then estimated by multivariable Cox regression models with the use of propensity-score inverse-probability weighting.

In addition to the comparisons using all patients, we compared the behavior of biomarkers in patients that received CP with propensity-matched controls obtained from the SOC group. Patients were matched in a 1:1 relation, using the nearest neighbor method considering the logit as the distance method. Maximum distance allowed was 0.10. At each matching, the unit with the closest logit still unmatched was used. After checking the balance of the propensity-matched groups, we compared sequential assessments of clinical and laboratory parameters.

Statistical tests were two-sided. A *p* < 0.05 was considered statistically significant. Statistical analyses were performed using IBM SPSS Statistics version 23.0 (Chicago, IL, USA), R (4.0.2) and Prism (GraphPad, San Diego, CA, USA).

## Results

### Characteristics and Organ Dysfunction at Admission

From March 17th to May 30th, 2020, 153 patients were admitted to our ICU, from which 113 had confirmed COVID-19 and were included in the analysis (Supplementary eFigure 1). Forty-one patients were treated with CP therapy and 72 received standard of care. Among CP-treated patients, 23 received 1 infusion and 18 received 2 infusions of CP. The median age of CP recipients was 58 (IQR 45–64) years, which was lower than the SOC group [63 (IQR 49–71) years; *p* = 0.048]. Table 1 also shows that 61% were male, and 86% were mechanically ventilated at admission. From those on MV, 9% underwent prone position and 41% needed neuromuscular blockade on the first day of ICU. Median SAPS 3 was somewhat lower in the treated cohort, as compared to all SOC patients [CP 62 (54–69) vs. SOC 67 (57–77), *p* = 0.06], while organ dysfunction, as measured by the SOFA score (19), was not different [CP 10 (7–12) vs. SOC 11 (8–13), *p* = 0.15]. Baseline ARDS severity was worse [Severe ARDS in CP 11 (27%) vs. SOC 12 (17%), *p* = 0.09] and vasopressor need was more frequent in the SOC group [CP 19 (46%) vs. SOC 49 (68%), *p* = 0.02].

**Table 1:** Clinical characteristics at admission to the ICU.

**Characteristics**	**All patients (*N* = 113)**	**Convalescent plasma (*N* = 41)**	**Standard of care (*N* = 72)**	***p*-value (CP vs. standard)**
Age	61 (48–68)	58 (45–64)	63 (49–71)	0.048
Sex–male	69 (61%)	26 (63)	43 (60%)	0.7
**Race**				0.5
*Caucasian*	62 (55%)	24 (59%)	38 (53%)	–
*Black*	11 (10%)	5 (12%)	6 (8%)	–
*Other*	40 (35%)	12 (29%)	28 (39%)	–
Frailty score	2 (1–3)	1 (1–3)	2 (1–3)	0.7
Frail	35 (31%)	13 (32%)	22 (31%)	0.9
**Respiratory support**				0.5
*Oxygen*	16 (14%)	7 (17%)	9 (13%)	–
*Mechanical ventilation*	97 (86%)	34 (83%)	63 (88%)	–
Prone position	10 (9%)	3 (7%)	7 (10%)	0.7
Neuromuscular blockade	46 (41%)	20 (49%)	26 (37%)	0.2
SAPS 3	64 (56–75)	62 (54–69)	67 (57–77)	0.06
SOFA total	11 (7–13)	10 (7–12)	11 (8–13)	0.15
*SOFA renal*	1 (0–3)	0 (0–2)	1 (0–3)	0.22
**ARDS severity**				0.09
*No ARDS*	22 (20%)	5 (12%)	17 (24%)	
*Mild*	13 (12%)	2 (5%)	11 (15%)	
*Moderate*	55 (49%)	23 (56%)	32 (44%)	
*Severe*	23 (20%)	11 (27%)	12 (17%)	
**Complications/Therapy**				
Vasopressor	68 (60%)	19 (46%)	49 (68%)	0.02
Steroid treatment for ARDS	11 (10%)	7 (17%)	4 (6%)	0.1
Hydrocortisone for shock	27 (24%)	13 (32%)	14 (19%)	0.14
Anticoagulation	79 (70%)	30 (73%)	49 (68%)	0.6
Thromboembolism	10 (9%)	5 (12%)	5 (7%)	0.6

*SAPS 3, Simplified Acute Physiology Score 3; SOFA, Sequential Organ Failure Assessment; CP, convalescent plasma; ARDS, acute respiratory distress syndrome; Steroid treatment for ARDS: Dexamethasone or Methilprednisolone; Anticoagulation includes prophylactic and therapeutic; Thromboembolism includes the clinical diagnosis of either arterial or venous thrombosis*.

Comparisons of disease duration showed that the time between symptom-onset and ICU admission was similar between CP and SOC patients [10 (8–14) vs. 9 (5–12), *p* = 0.07]. On average, patients treated with CP received the first infusion on median day 1 (IQR 1–3) from admission and the second infusion on median day 6 (IQR 5–9) (Supplementary eTable 2).

Moreover, approximately half of patients in our cohort had hypertension, 31% had diabetes mellitus and 19% were obese. Other than obesity, which was non-significantly more common among CP-treated patients [11 (27%) vs. 10 (14%), *p* = 0.08], the distribution of comorbidities, as well as presenting symptoms, were similar between groups (Supplementary eTable 2). Baseline laboratory parameters showed no significant differences between CP and SOC patients (Supplementary eTable 1). Amongst 33 patients in which IgG titers were measured before CP administration, only 4 had endpoint titers < 1:1,080, and two equal to 1:1,080 (Supplementary eTable 2). These results were not available prior to CP administration.

### Clinical Improvement and Survival at 28 Days

**Figure 1:**
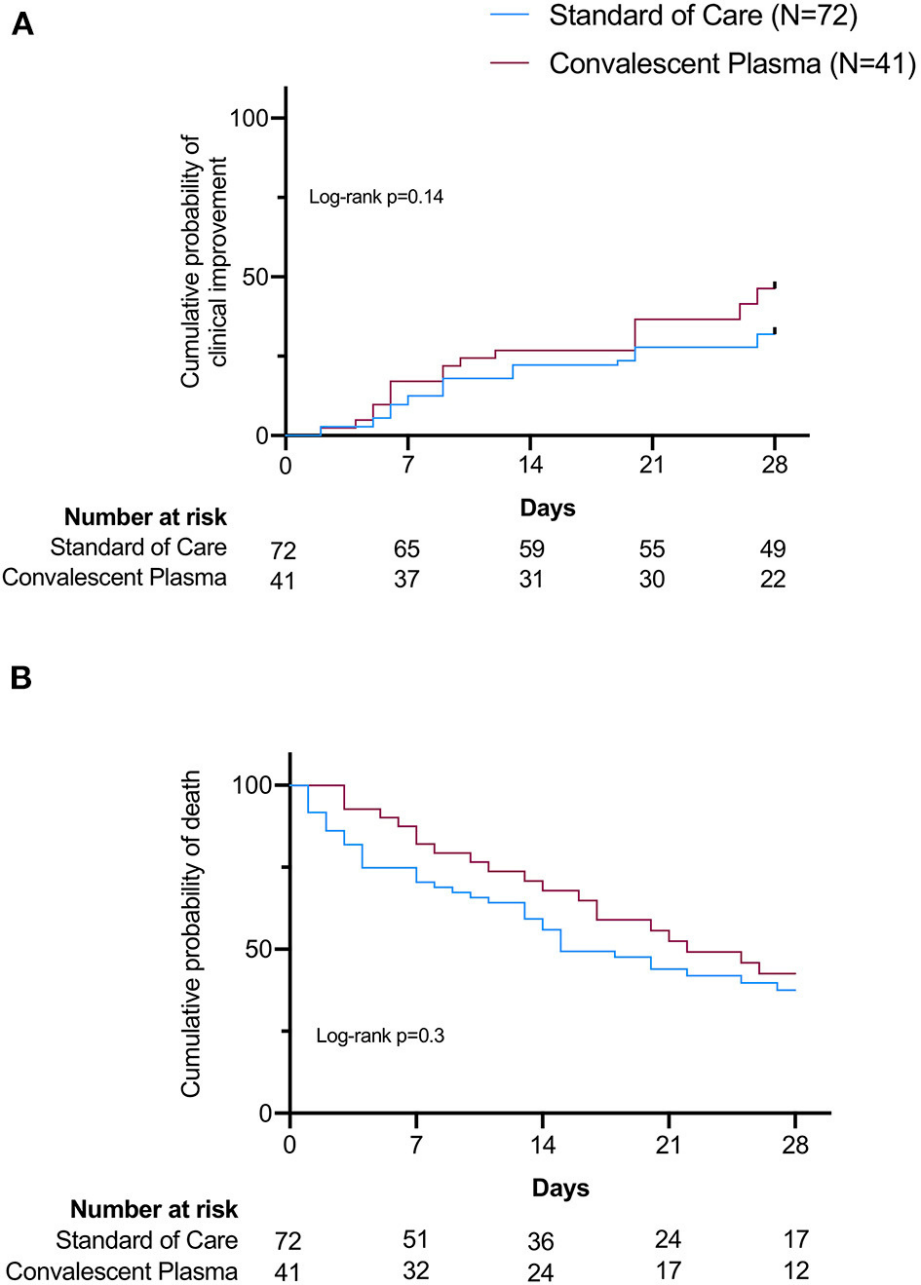
Univariable survival curves (Kaplan–Meier) of 28-day outcomes: Probability of clinical improvement **(A)** and survival **(B)** in Standard of Care (SOC) and Convalescent Plasma (CP) groups. Differences among curves were assessed using the log-rank test with a confidence level of 0.05.

**Figure 2:**
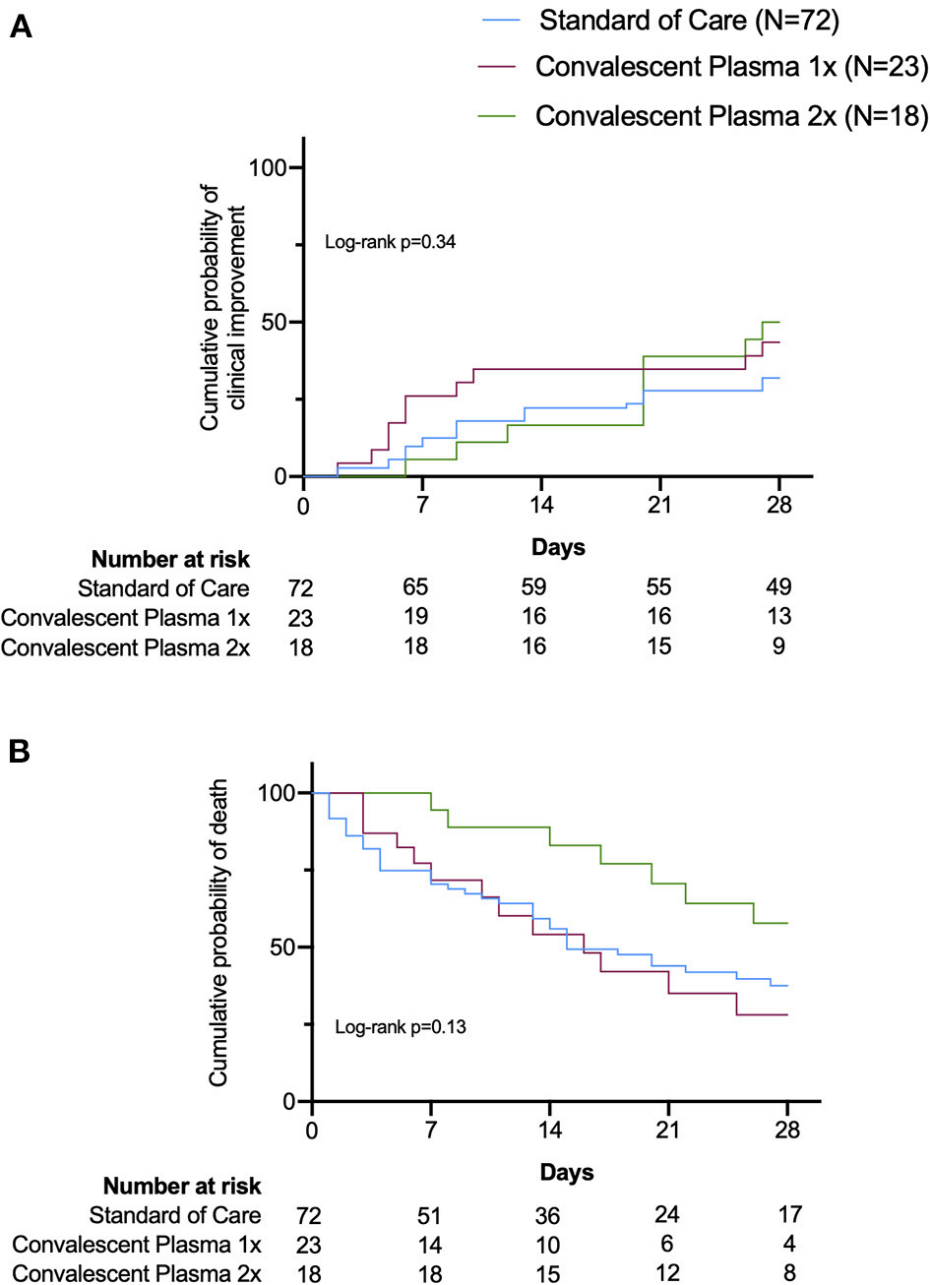
Univariable survival curves (Kaplan–Meier) of 28-day outcomes: Probability of clinical improvement **(A)** and survival **(B)** in Standard of Care (SOC), 1 infusion of Convalescent Plasma (CP) and 2 infusions of CP groups. Differences among curves were assessed using the log-rank test with a confidence level of 0.05.

Clinical improvement within 28 days was achieved in 19 patients in the CP group (46%) and in 23 patients in the SOC group (32%). Univariate Kaplan–Meier (KM) curves showed no significant differences in the probability of clinical improvement between groups (Figure 1A, Log-rank *p* = 0.14). Mortality rates, assessed at 7 and 28 days, were 17 and 49% in the CP group, and 29 and 56% in the SOC, respectively (Table 2). KM curves showed no significant differences in survival to 28 days between SOC and CP-treated patients (Figure 1B, Log-rank *p* = 0.3). When analyzing 1-time and 2-times CP infusions separately, the KM curves also suggested no significant benefit of either treatment strategy on clinical improvement or survival (Figures 2A,B, Log-rank *p* = 0.34 and *p* = 13, respectively).

**Table 2:** Outcomes.

**Outcomes**	**All patients (*N* = 113)**	**Convalescent plasma (*N* = 41)**	**Standard of care (*N* = 72)**	***p*-value (CP vs. standard)**
Hospital length of stay (at 28 days)	16 (6–28)	17 (7–28)	14 (4–26)	0.16
Ventilator free days (at 28 days)	0 (0–6)	0 (0–8)	0 (0–3)	0.24
Clinical improvement	42 (37%)	19 (46%)	23 (32%)	0.13
Mortality at 7 days	28 (25%)	7 (17%)	21 (29%)	0.15
Mortality at 21 days	54 (48%)	17 (42%)	37 (51%)	0.3
Mortality at 28 days	60 (53%)	20 (49%)	40 (56%)	0.5
**Outcomes in subgroup with moderate to severe ARDS**	(*N* = 78)	(*N* = 34)	(*N* = 44)	
Hospital length of stay (at 28 days)	17 (6–28)	20 (10–28)	14 (4–27)	0.05
Clinical improvement	22 (28%)	12 (35%)	10 (23%)	0.2
Mortality at 7 days	25 (32%)	7 (21%)	18 (41%)	0.06
Mortality at 21 days	45 (57%)	17 (50%)	28 (64%)	0.2
Mortality at 28 days	49 (63%)	20 (59%)	29 (66%)	0.5

**Figure 3:**
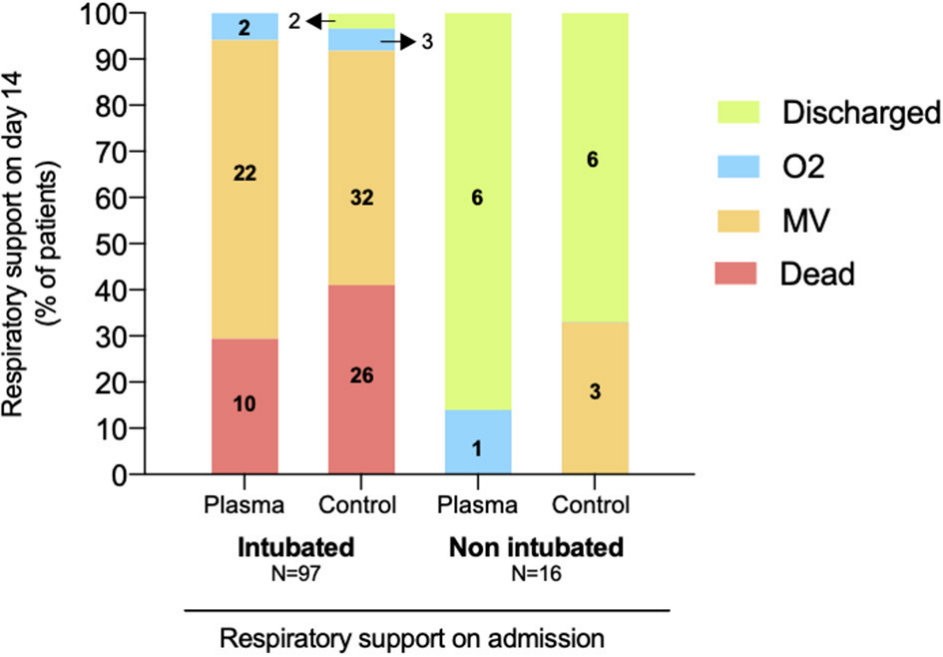
Clinical status and respiratory support on day 14 after admission, according to admission status. Columns represent patients in the Convalescent Plasma and Standard of Care groups, separated by their intubated and non-intubated status at admission to the ICU. Colors represent percentages and numbers within colors represent the *N* of patients in each subgroup.

The 14-day evolution of intubated and non-intubated patients from both groups is demonstrated in Figure 3. Twenty six out of 63 (41%) patients that were intubated at admission died within 14 days in the SOC group, as compared to 10 out of 34 (29%) in the CP group. Among those not requiring early invasive ventilation, 3 out of 9 in the SOC group were intubated in the first 2 weeks of ICU, while none required MV in the CP group.

Additionally, we built two multivariable (MV) models with the primary outcomes above, using inverse-probability propensity score weighting, and adjusting for age, mechanical ventilation, SOFA score, SAPS 3, frailty and time from symptom onset to ICU admission. In MV analyses, CP was not independently associated with clinical improvement [adjusted Hazard Ratio (aHR) 0.91 (0.49–1.69)] or 28-day mortality [aHR 0.90 (0.52–1.57)].

### Sequential Analyses of Biomarkers

**Figure 4:**
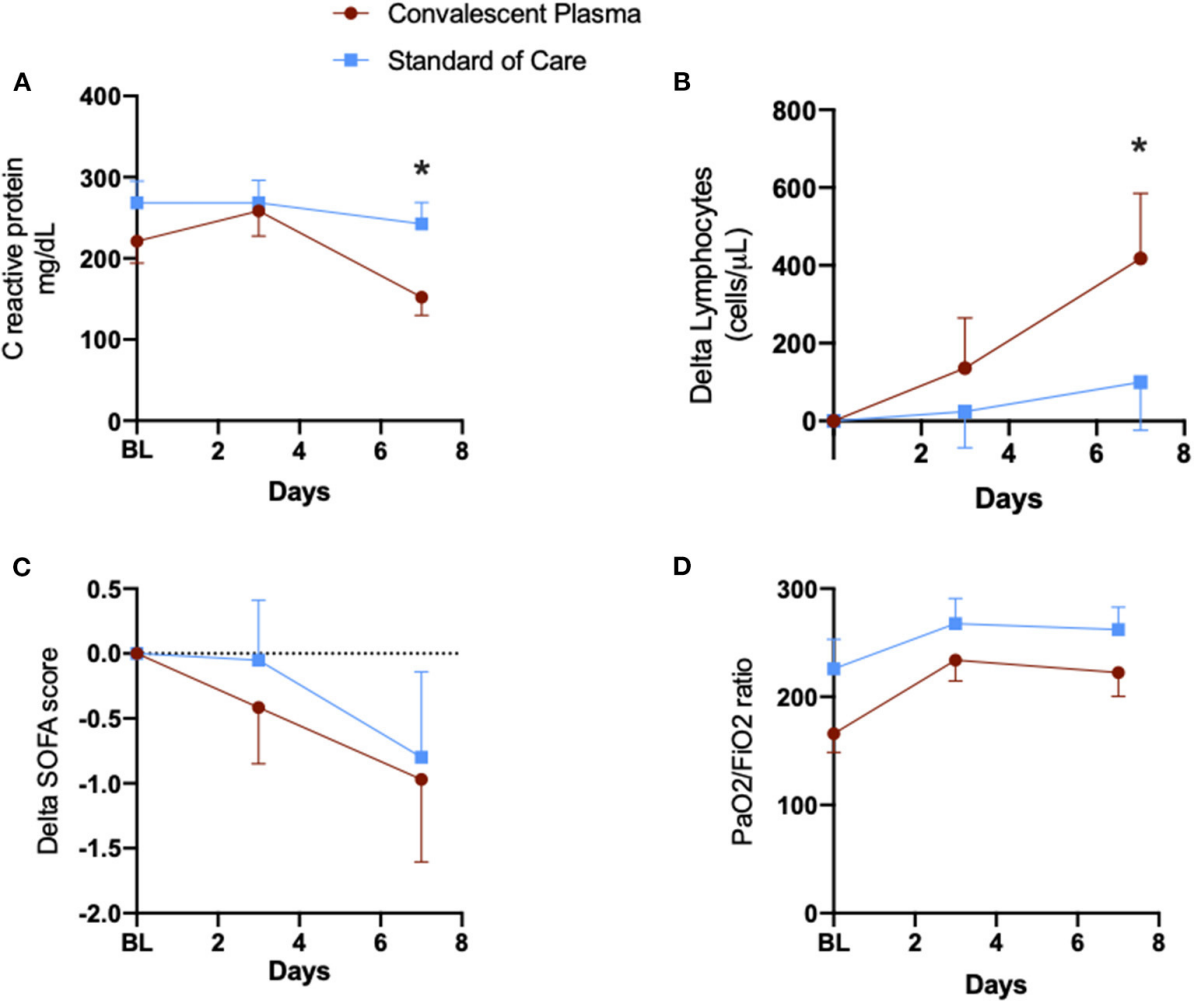
Temporal changes of clinical and laboratory parameters in patients that received convalescent plasma and propensity-matched controls. Data are expressed as mean (standard error of the mean). *corresponds to *p* < 0.05. BL, baseline; SOFA, sequential organ failure assessment; Delta values represent the change from baseline. **(A)** C reactive protein. **(B)** Delta lymphocytes. **(C)** Delta SOFA score. **(D)** PaO2/FiO2 ratio.

Temporal changes of biomarkers demonstrated a significant reduction of C-reactive protein (CRP) on day 7 in the CP group [mean (SE) CPR in CP 152.2 (22.5) mg/dL vs. Control 242.5 (26.4) mg/dL, *p* = 0.03] and a significant increase in the lymphocyte count, also on day 7 [mean (SE) delta lymphocyte difference of +418.1 (167.1) in CP vs. +99.7 (123.3) in Controls, *p* = 0.04], as compared to PS-matched patients in the SOC group (Figures 4A,B). There were no significant changes in pulmonary oxygen exchange, as measured by the PaO2/FiO2 ratio or multiorgan dysfunction, as measured by changes in the SOFA score (Figures 4C,D). Moreover, the plasma cytokine IP-10 showed progressive reductions in the first week after CP, while IL-6 remained unchanged from baseline (Supplementary eFigure 2).

Quantitative viral loads were assessed in respiratory secretions of patients treated with CP. The rates of negative SARS-CoV-2 viral PCR among those treated with one infusion of CP was 14 and 21% after 3 and 7 days, respectively. Patients that received two infusions had their viral load assessed up to 14 days. In this group, all had detectable virus on days 3, 7, and 10. At 14 days after infusion, the rate of negative PCR was 46% (Supplementary eFigure 3).

### Convalescent Plasma Safety

We did not identify any adverse effects imputable to CP transfusion in the 59 transfused doses.

## Discussion

In this observational study of critically ill patients with COVID-19, we found no significant differences in the time to clinical improvement or survival to 28 days between patients treated with convalescent plasma therapy and those who received standard of care treatment. We further observed anti-inflammatory and lymphocyte-recovery effects in patients treated with CP, as compared to matched controls.

Subgroup analyses of a previous clinical trial and results of an observational study suggested clinical benefit of CP in patients with severe COVID-19, but not in those mechanically ventilated (i.e., life-threatening disease) (10, 20). This was not confirmed by a more recent publication, where patients with pneumonia and hypoxemia were randomized to receive CP or placebo, which showed no clinical improvement or reduced mortality in the treated group, as compared to controls (21). In our study, we observed that invasive ventilation was avoided in all patients in the CP group without moderate-to-severe ARDS in our cohort (*N* = 7) and they survived to 28 days. In comparison, in the SOC group, 11 out of 28 without moderate-to-severe ARDS (39%) died within 28 days. Challenging this hypothesis that earlier administration of CP (before advanced pulmonary dysfunction) would more likely improve outcomes, an open label randomized trial including patients with early moderate disease showed no difference in the composite outcome of progression of pulmonary injury or death (15). However, 86% of CP-treated patients in the trial had measurable neutralizing antibodies. Combined, these findings suggest that more data is needed on patients with early disease and absent antibody response to define the efficacy of CP for COVID-19.

Our findings demonstrated that CRP levels were significantly reduced and lymphocyte counts increased 7 days after CP administration, as compared to matched controls. Inflammatory cytokine IP-10 also reduced progressively in CP-treated patients. Multiple organ dysfunction, as measured by the SOFA score, and pulmonary gas exchange, as measured by the PaO2/FiO2 ratio, were not different between groups. CRP, IP-10 and lymphocyte count have been associated with severity of clinical presentation and temporal changes of these biomarkers have been reported in patients with clinical improvement and better outcomes (22–26). Previous case series of patients that received CP showed reduced CRP levels and SOFA scores, as well as improved PaO2/FiO2 ratios, but no comparisons with untreated patients were available to discriminate the effect of the treatment from the natural history of the disease. The positive physiological effects of CP, even in IgG positive patients, could be due to neutralizing antibodies in donated plasma, although we did not measure it in our study.

In contrast to published case series and a randomized trial, that showed undetectable viral PCR early after CP in more than 80% of treated patients (10, 13), only 21% of our patients showed viral clearance in respiratory secretions after only one infusion of CP. Among those that received 2 CP doses, 46% had negative PCR as late as 14 days after infusion. These differences may seem promising for higher doses of CP, but the fact that we did not measure sequential PCR in the SOC group limits the interpretation of these findings. In comparison to previous studies, CP-related factors were similar, including plasma volume administered and anti-S antibody concentrations (10, 13). One potential explanation for the smaller rate of viral clearance in our patients is that they were treated earlier in the course of the disease. Median time from symptom-onset to CP was 13 (IQR 9–17) days in this study vs. 30 and 45 days in previous reports (10, 13). Based on previous work, virus persistence has been linked to severe disease and poor outcome (2). In addition, promising therapies for COVID-19 showed faster decline in viral load in treated vs. control groups (10, 27) or progressive reductions in uncontrolled studies (7–9). Thus, the inability of CP therapy to promote viral clearance may reflect the severity of our study population, which may have limited any potential benefit of CP.

Although plasma transfusion can lead to adverse events, we did not observe either mild or serious transfusion-related complications in our cohort, despite active surveillance. This is in line with a recently published large cohort of COVID-19 patients receiving CP, where serious adverse events were found in <1% of recipients (28).

This study has limitations. First, its non-randomized design and small sample size limited definite conclusions on the clinical benefit of CP. Second, the majority of CP-treated patients had high baseline anti-S antibody concentrations. Although there is controversy in this topic, this may have limited the main benefit associated with CP therapy, namely the improvement in immunological response (11). Third, standard of care support has evolved along the COVID-19 pandemic. Our cohort, treated between March and May, 2020, did not receive protocolized steroid treatment, which is currently considered the evidence-based for patients with COVID-19 and hypoxemia, pneumonia or ARDS. Finally, patients in the standard of care group did not undergo sequential viral load assessments, which precluded a comparative analysis of viral clearance.

## Conclusions

In summary, convalescent plasma therapy showed a non-significant reduction in short-term mortality, but was not associated with clinical improvement or survival at 28 days. These results may be explained by our small sample size, the inclusion of patients with life-threatening disease, and elevated baseline IgG titers. These findings may guide future trials to identify patients with early disease and without antibody response that may benefit from CP therapy.

## Data Availability Statement

The raw data supporting the conclusions of this article will be made available by the authors, without undue reservation.

## Ethics Statement

The studies involving human participants were reviewed and approved by Brazilian Registry of Clinical Trials (ReBEC) with the identification RBR-4vm3yy. The patients/participants provided their written informed consent to participate in this study.

## Author Contributions

PK, CR, MG, NC, FF, RT, BG, AG, RL, CA, VC, MAP, NV, CG, FM, and PJ worked directly with patient and lab analysis. MP, SM, ML, SC, ÁO, AR, JL, and ON worked with many steps of plasma donation. AV, LH, and FLM worked with anti-S IgG titration and PRNT. LC worked in the S protein expression. FB and PB worked in the cytokines measurement. PK, CR, MG, FB, LB, OF, AT, PF, and LA participated in this work design, discussion of results, and manuscript preparation. All authors contributed to the article and approved the submitted version.

## Conflict of Interest

The authors declare that the research was conducted in the absence of any commercial or financial relationships that could be construed as a potential conflict of interest.

## Footnotes

^1^ISARIC (2020).
