# Supplementary material for: Effect of Convalescent Plasma in Critically Ill Patients With COVID-19: An Observational Study
Source: Front Med (Lausanne). 2021 Jan 28;8:630982. doi: 10.3389/fmed.2021.630982 (PMC7876445; doi:10.3389/fmed.2021.630982)
Supplement: Supplementary file 1 [file Data_Sheet_1.PDF]

Additional Files - Supplemental Material

METHODS

Convalescent Plasma Donors

Convalescent plasma donors were recruited and screened as described (Supp). Inclusion criteria for donation were age between 18 and 60 years, previous RT-PCR evidence of SARS-CoV-2 infection, absence of symptoms for at least 14 days, negative anti-HLA antibody for female candidates and negative RT-PCR for SARS-CoV-2 in plasma at the time of recruitment. Additionally, donors were required to present IgG anti-SARS-CoV-2 antibodies with reactivity on immunochromatographic qualitative test (Medtest, Yuhand district, China) plus a titer equal to or higher than 1:1080 serial dilutions, using an anti-Spike Enzyme-Linked Immunosorbent Assay (ELISA). After donation, CP underwent pathogen inactivation with Intercept® kits (Cerus Corporation, Amersfoort, The Netherlands).

1. Inclusion Criteria:

Inclusion criteria for plasma donors
Age equal or over 18 and under 60
Confirmed COVID-19 with laboratory evidence showing that they were infected by SARS-CoV-2
No COVID-19 symptoms for at least 14 days.
Negative anti-HLA antibody screening for female candidates with one or more previous pregnancy
Negative molecular test (RT-PCR) for SARS-COV-2 in plasma
Compliance with the eligibility criteria for blood donors (including specific criteria for apheresis plasma donors), described in the decree 5/2017, Ministry of Health - except for recent SARS-CoV-2 infection.

Presence, in serum or plasma, of IgG anti-SARS-CoV-2 antibodies, with a titer equal to or higher than 80.
Negative infectious disease markers (HBsAg, anti-HBc, anti-HCV, anti-HIV1+2, anti-HTLV-I/II, syphilis and Chagas), by chemoluminescence, and negative NAT tests for HIV, HCV and HBV.
Signature of an informed consent form
Exclusion criteria
Pregnancy
Refusal to sign informed consent

2. Assessment and Procedures in Plasma Donors

The candidate who accepted the invitation from HEMORIO was interviewed by one of the researchers, who carried out donor evaluation, explained the project and collected the informed consent signature. If the candidate was apt for plasma donation, venous blood samples were collected to perform the tests described below.

2.a. Tests on prospective donor samples:

- Class I and class II anti-HLA antibodies screening in plasma by using a Luminex®xMAP® analyser (Luminex Corporation, Texas, USA). for non-nulliparous women
- RT-PCR testing for SARS-CoV-2 in plasma was carried out in plasma from all prospective donors (Molecular IDT Integrated DNA Technologies SARS-CoV-2 – N1/N2/P, Promega, Madison, USA). The tests were performed according to the manufacturer´s instructions, using the MDX Instrument and kits (Qiagen, Hilden, Germany) for RNA extraction and Applied Biosystem MDX thermocycler instrument (Thermo-Fisher, Waltham, USA).
- ABO and Rh donor typing and anti-erythrocyte antibody screening, using microplaque technique and Erythra instrument from Grifols (Barcelona, Spain).
- Infectious diseases marker tests for HIV, HBV, HCV, HTLV, syphilis and Chagas disease - by ELISA (Diasorin, Turin, Italy) and chemoluminescence (Abbott, Chicago, USA).
  Anti-A and anti-B agglutinin titration by gel-test method (Grifols, Barcelona, Spain)

- Anti-SARS-CoV-2 IgG and IgM antibody detection, using the immunochromatographic rapid test MedTest Coronavirus 2019-nCoV IgG/IgM, from MedLevensohn manufacturer (Yuhang District, China). The reaction intensity was classified in 1+ to 4+. We performed the tests in serum, according to the manufacturer's instructions. If IgG was positive, then we performed a titration using the anti-Spike Enzyme-Linked Immunosorbent Assay (ELISA).

2.b. Analysis of test results

The day after (or up to seven days after) the prospective donor´s visit, the test results were obtained; if the results met the inclusion criteria, donors were invited to return to HEMORIO, at the earliest possible date, to donate plasma.

2.c. Plasma collection and testing

600 ml of plasma were collected, by apheresis, in the MCS 3-Plus cell separator machine, from Haemonetics (Braintree, USA), using the institution's regular procedure for non-therapeutic plasmapheresis.

In a sample taken at the time of plasma donation, the following tests were repeated:
- Infectious disease marker tests
- NAT
- ABO and Rh typing
- Anti-erythrocyte antibody screening

2.d. Pathogen Inactivation

Pathogen inactivation was performed using Intercept® kits, manufactured by Cerus Corporation (Amerfoort, The Netherlands) according to manufacturer´s instructions. The pathogen inactivation was based on Psoralen + Ultraviolet-A irradiation pathogen photo-inactivation.

Molecular Methods:

1. **Real-time Reverse-Transcription Polymerase Chain Reaction Assays**

In all patients, to diagnose SARS-CoV-02 infection, RT-qPCR for SARS-CoV-02 RNA was performed on lower respiratory tract aspirates collected upon admission. Total nucleic acid from clinical samples was extracted using Maxwell® RSC Viral TNA kit (Promega, Madison, USA) as instructed by the manufacturer. For all specimens, 200 μL of sample was used for RNA extraction, and extracted RNA was finally eluted in 50 μL of RNase-free water and stored at –80°C. The RT-qPCR was performed using a commercial kit specific for 2019-nCoV detection (Integrated DNA Technologies - IDT, Iowa, USA) approved by the USA Centers for Disease Control and Prevention (CDC)(1). In a typical reaction, 5 μL of RNA was amplified in a 20-μL reaction containing 0.4 μL GoScript™ RT Mix for 1-Step RT-qPCR 50x (Promega), 9.98 μL of GoTaq® qPCR Master Mix 2x (with CXR Reference Dye), 0.02 μL of CXR Reference Dye, 3.1 μL nuclease-free water, and 1.5 μL primers-probe mixture (Assays by Design, Integrated DNA Technologies – IDT). Plasmids containing the target sequences were used as standard controls. All samples were tested for the endogenous RNAse P gene as an internal control.

All RT-qPCR reactions were performed on QuantStudio™ 7 Flex Real-Time PCR System (Applied Biosystems, Foster, CA, USA). Cycling parameters were set as: 45°C for 15 minutes for reverse transcription, followed by 95°C for 2 minutes and then 45 cycles of 95°C for 3 seconds and 55°C for 30 seconds. RT-qPCR assay provided a cycle threshold (Ct) value, which is the number of cycles necessary for the fluorescent signal to cross the threshold. For each sample, three targets were evaluated (N1,N2 and RP) and a sample was considered positive for the novel coronavirus if both N1 and N2 had a Ct value up to 40. If the Ct (N1 and N2) was undetectable, the specimen was considered negative. When only one of the two targets (N1 or N2) was positive (< 40 Ct) the result was considered inconclusive. If an initial result was inconclusive, the extracted RNA was retested for RT-qPCR, if both tests were inconclusive, the final result was reported as inconclusive.

Before and after convalescent plasma therapy, lower respiratory tract aspirates and plasma samples were collected and the SARS-CoV-2 RNA was monitored. All serial

samples were stored at –80°C and then processed at a single time. The Ct values of recipients for targets N1 and RP were obtained on baseline, day 1, day 2, day 3, day 4, day 5, day 6, and day 7 after convalescent plasma therapy. RNase P Ct values were used to correct N1 Ct values for each sequential samples of each individual used the following formula: N1 Ct_{sample} – (RNase P Ct_{sample} – RNase P $Ct_{pre\text{-}treatment}$). In this case the RNase P Ct of pre-treatment was used as reference.

RESULTS

Supplemental Tables and Figures

eTable 1. Clinical characteristics at admission to the ICU and outcomes of patients that received convalescent plasma and propensity score-matched controls that received standard of care

**Characteristics**	**Convalescent Plasma (N=41)**	**PS Matched Controls (N=41)**
Age	58 (45 – 64)	56 (45 – 65)
Sex – Male	26 (63)	28 (68%)
**Race**		
Caucasian	24 (59%)	23 (56%)
Black	5 (12%)	2 (5%)
Other	12 (29%)	12 (27%)
Frailty Score	1 (1 – 3)	1 (1 – 3)
Frail	13 (32%)	13 (32%)
**Respiratory support**		
Oxygen	7 (17%)	4 (17%)
Mechanical ventilation	34 (83%)	34 (83%)
Prone position	3 (7%)	3 (8%)
Neuromuscular blockade	20 (49%)	14 (35%)
Vasopressor	19 (46%)	25 (61%)
SAPS 3	62 (54 – 69)	63 (54 – 73)
**SOFA total**	10 (7 – 12)	11 (7 – 13)
SOFA renal	0 (0 – 2)	1 (0 – 2)
**ARDS severity**		
No ARDS	5 (12%)	10 (24%)
Mild	2 (5%)	7 (17%)
Moderate	23 (56%)	19 (46%)
Severe	11 (27%)	5 (12%)
Vasopressor	19 (46%)	25 (61%)
**Outcomes**		
Mortality at 7 days	7 (17%)	4 (10%)
Mortality at 21 days	17 (42%)	17 (42%)
Mortality at 28 days	20 (49%)	20 (49%)

PS, propensity score; PSMC, propensity score matched controls; SAPS 3, Simplified Acute Physiology Score 3; SOFA, Sequential Organ Failure Assessment; CP, convalescent plasma

eTable 2. Baseline signs, symptoms, and comorbidities

**Comorbidities**	**All Patients (N=113)**	**Convalescent Plasma (N=41)**	**Standard of Care (N=72)**
Obesity	21 (19%)	11 (27%)	10 (14%)
Hypertension	60 (53%)	22 (54%)	38 (54%)
Diabetes	35 (31%)	12 (29%)	23 (32%)
Cancer	5 (4%)	1 (2%)	4 (6%)
Coronary artery disease	3 (3%)	1 (2%)	2 (3%)
Congestive Heart Failure	5 (4%)	3 (8%)	2 (3%)
Any cardiac disease	7 (6%)	4 (10%)	3 (4%)
COPD	5 (4%)	0	5 (7%)
Neurological disease	3 (3%)	2 (5%)	1 (1%)
Chronic kidney disease	3 (3%)	1 (2%)	2 (3%)
Chronic steroid use	1 (1%)	1 (1%)	0 (0%)
**Disease duration**			
Days from symptom onset to admission	10 (6 – 13)	10 (8 – 14)	9 (5 – 12)
Days from symptom onset to Convalescent Plasma	-	13 (9 – 17)	-
Days from admission to CP (First infusion) (N=41)	-	1 (1 – 3)	-
Days from admission to CP (Second infusion) (N=18)	-	6 (5 – 9)	-
**Baseline Anti-S IgG titers**		**(N = 33)**	
< 1:1,080		4	
= 1:1,080		2	
> 1:1,080		27	
**Presenting symptoms**			
Cough	84 (74%)	31 (76%)	53 (75%)
Fever	99 (88%)	36 (88%)	63 (88%)
Dyspnea	94 (83%)	36 (88%)	58 (82%)
Fatigue	52 (46%)	20 (49%)	32 (44%)
Headache	16 (14%)	6 (15%)	10 (14%)
Anosmia	21 (19%)	9 (22%)	12 (17%)
Altered mental status	58 (51%)	22 (54%)	36 (50%)

CP, convalescent plasma; COPD, chronic obstructive pulmonary disease.

eTable 3. Baseline blood count and biochemical markers

Laboratory values	All Patients (N=113)	Convalescent Plasma (N=41)	Standard of Care (N=72)	p value (CP vs Standard)	PS Matched Controls (N=41)	p value (CP vs PSMC)
White blood cell count	15.9 (11.6 – 21)	14.2 (10.4 – 25.8)	15.9 (12.1 – 19.7)	0.8	14.6 (11.6 – 18.6)	0.5
Lymphocyte count (x1,000)	1.22 (0.75 – 1.78)	1.09 (0.68 – 1.60)	1.30 (0.86 – 1.85)	0.2	1.29 (0.86 – 1.73)	0.4
Hemoglobin	11.8 (10.2 – 12.8)	11.8 (10.3 – 12.8)	11.8 (10.3 – 12.8)	0.8	12 (11 – 13.2)	0.2
Platelet count	185 (131 – 239)	184 (154 – 226)	186 (129 – 242)	0.9	182 (126 – 244)	0.7
C Reactive Protein	230 (94 – 386)	198 (73 – 351)	259 (153 – 410)	0.1	236 (154 – 373)	0.1
Creatinine	1.4 (0.9 – 3.5)	1.2 (0.8 – 2.6)	1.5 (1 – 3.7)	0.06	1.3 (1 – 2.4)	0.3
Arterial Lactate (N=86)	1.6 (1.3 – 2.4)	1.6 (1.4 – 2.2)	1.6 (1.2 – 2.4)	0.7	1.4 (1.2 – 2)	0.1
pH	7.3 (7.2 – 7.4)	7.3 (7.2 – 7.4)	7.3 (7.2 – 7.4)	0.5	7.3 (7.2 – 7.4)	1
Base excess	-0.2 (-5.1 – 3.8)	0.5 (-2.2 – 4.5)	-0.4 (-6.3 – 3.6)	0.08	0.7 (-4.6 – 3.9)	0.5
AST (N=91)	52 (31 – 83)	35 (30 – 62)	69 (35 – 103)	0.02	48 (29 – 82)	0.4

PS, propensity score; PSMC, propensity score matched controls; CP, convalescent plasma; AST, aspartate aminotransferase.

eTable 4. Proportion of patients in which SARS-CoV-2 RNA in respiratory secretions decreased to an undetectable level in those that received one or two infusions of Convalescent Plasma (CP).

Time after CP	Single CP Infusion (N=14)	Two CP Infusions (N=15)
3 days after infusion	2 (14%)	0
7 days after infusion	3 (21%)	0
10 days after infusion	Not measured	0
14 days after infusion	Not measured	7 (46%)

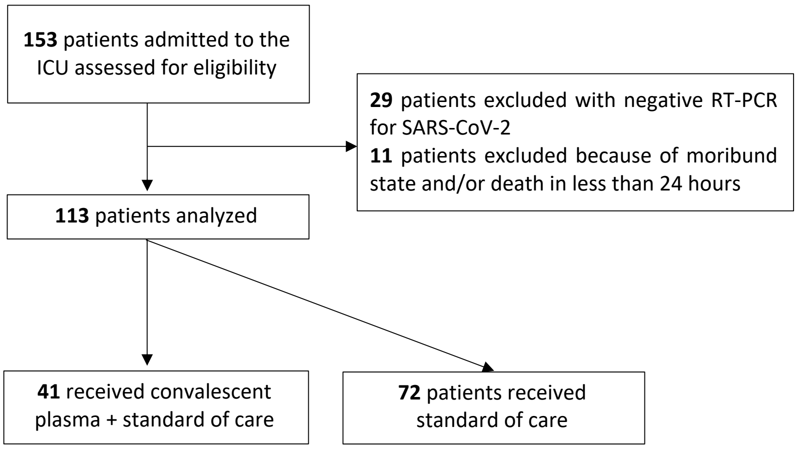

eFigure 1. Study flow diagram

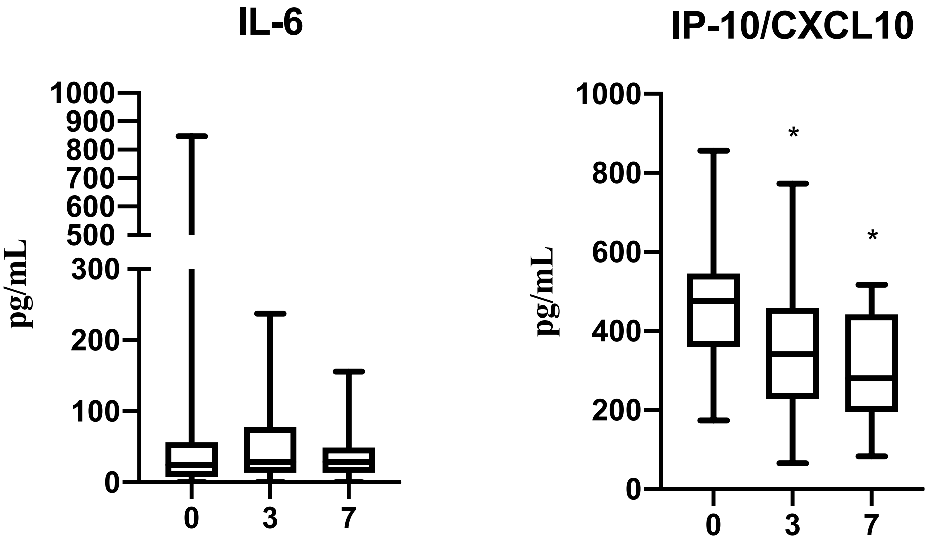

eFigure 2. Temporal changes in plasma cytokine concentrations of interleukin (IL)-6 and IFN-γ–induced protein 10 (IP-10) in patients that received convalescent plasma (CP) (N=XX). Data are expressed as boxplots with median and interquartile range. * corresponds to $p<0.05$. 0, baseline before CP; 3, 3 days after CP; 7, 7 days after CP.

References

1. Prevention CfDCa. CDC 2019-Novel Coronavirus (2019-nCoV) Real-Time RT-PCR Diagnostic Panel For Emergency Use Only Instructions for Use. 2020.
